# Editorial

**Published:** 1915-04

**Authors:** 


### EDITORIAL

The Business Office of the *Journal-Record* is 23 West Alabama Street.

The Editorial Office is 1014-15 Atlanta National Bank Building.

Address all Business Communications to *Journal-Record of Medicine*, 23 West Alabama Street.

Make remittances payable to *The Journal-Record of Medicine.*

On matters pertaining to the Editorial and Original Communications, address Edgar G. Ballenger, M. D., Atlanta, Ga.

Reprints of Original Articles will be furnished at cost price. Requests for the same should always be made in THE MANUSCRIPT.

We will present, postpaid, on request to each contributor of an original article, twenty (20) marked copies of *The Journal-Record of Medicine* containing such article.

### TUBERCULOSIS-REINFECTION.

The campaign of education concerning tuberculosis that has been waging in the principal cities of the United States during the last decade has been effective in many good ways, but it seems to be reaching the end of its rope so far as any educational matter might be thought to have an end. The smallest child knows enough of the disease to be found telling its baby brother something about it and warning him according to the pictures displayed and the maxims set forth in the kindergartens of the dangers of promiscuity, dirt and lack of ventilation and sunlight. The oldest adult sets aside the habits of age for the time, ceases to say "We never did this when I was a boy," and endeavors to conduct himself according to the rules of health as he understands them in the prevention of tuberculosis. The general condition has been improved and the minds of the people are open to instruction but the disease continues its fatal sway without apparent abatement.

Statistics are unreliable because there have been much of concealment and lack of accuracy in the registration of tuberculosis. One has to depend upon his own observation if he has come into contact with many cases and to seek information from places where the records have been analyzed by the men who made them.

It is our observation that during the past two years, there has been little or no gain in the fight against the disease.

Dr. Bang, writing in the London Lancet, points out that in Copenhagen, many early cases that had been discharged symptom free had recurrences later on and that a large number of more advanced cases that had been discharged as unimproved and likely to fail rapidly, ultimately improved and a few became self-supporting . He explains part of this by showing that the classification is faulty and as for the rest, he urges that third stage cases be given longer residence and greater attention in the sanitoriums.

It all harks back to the fact that we have constantly living in every community many cases of tuberculosis in the ordinary intimacy of life and that we are vainly fighting to avoid the very dangers we are cultivating.

We welcome the return of the improved or cured case of tuberculosis from the hospital although we know that there is grave risk to ourselves and to him in doing so . We know that many many men who return home have to be sent back or be cared for in their homes. We know, if we care to ask ourselves the question, that the reason why they have relapses is two-fold. One that they have not been improved at all but are simply in a temporary, quiescent state, and the other that they have themselves, become the victims of Reinfection. We know that improved cases seem to get well and stay well if they go to certain climates and neighborhoods and we have ascribed virtues to these places when as a matter of fact the main benefit the sufferer derived from the change was the fact that he went to so sparsely settled a place or one so well managed hygienically that he was not exposed to reinfection.

The cases that return to sizable communities and take up the ordinary life, die of tuberculosis and before they die, they start many others upon the same fatal track.

The time has come when we should add to our system of education all the emphasis we can express that the sufferers with tuberculosis should be isolated from the rest of the people and kept so. A public sentiment should be built up and eventually formulated into law that all cases of tuberculosis should be isolated and kept so with pleasant surroundings but under strictest quarantine until they were recovered. Then there would be no more reinfection of so-called well cases and what is better there would be no more tuberculosis.

If this sounds utopian or millenial, it is pleasanter than the contemplation of the horrible state of affairs at present existing. It is as practicable as any other big thing such as cleaning up the Isthmus or shooting a hundred thousand strong men a day or any of a dozen other things we are occupying our attention with these times. R. R. D.

